# Shared sanitation facilities and risk of respiratory virus transmission in resource‐poor settings: A COVID‐19 modeling case study

**DOI:** 10.1111/risa.17633

**Published:** 2024-08-23

**Authors:** Michael A. L. Hayashi, Sophia M. Simon, Kaiyue Zou, Hannah Van Wyk, Mondal Hasan Zahid, Joseph N. S. Eisenberg, Matthew C. Freeman

**Affiliations:** ^1^ Department of Epidemiology, School of Public Health University of Michigan Ann Arbor Michigan USA; ^2^ Gangarosa Department of Environmental Health, Rollins School of Public Health Emory University Atlanta Georgia USA; ^3^ Department of Environmental Science and Policy University of California Davis California USA; ^4^ Department of Epidemiology, Bloomberg School of Public Health Johns Hopkins University Baltimore Maryland USA

**Keywords:** individual‐based transmission model, infrastructure, mathematical model, respiratory pathogens, sanitation, WASH

## Abstract

Water supply and sanitation are essential household services frequently shared in resource‐poor settings. Shared sanitation can increase the risk of enteric pathogen transmission due to suboptimal cleanliness of facilities used by large numbers of individuals. It also can potentially increase the risk of respiratory disease transmission. As sanitation is an essential need, shared sanitation facilities may act as important respiratory pathogen transmission venues even with strict control measures such as stay‐at‐home recommendations in place. This analysis explores how behavioral and infrastructural conditions surrounding shared sanitation may individually and interactively influence respiratory pathogen transmission. We developed an individual‐based community transmission model using COVID‐19 as a motivating example parameterized from empirical literature to explore how transmission in shared latrines interacts with transmission at the community level. We explored mitigation strategies, including infrastructural and behavioral interventions. Our review of empirical literature confirms that shared sanitation venues in resource‐poor settings are relatively small with poor ventilation and high use patterns. In these contexts, shared sanitation facilities may act as strong drivers of respiratory disease transmission, especially in areas reliant on shared facilities. Decreasing dependence on shared latrines was most effective at attenuating sanitation‐associated transmission. Improvements to latrine ventilation and handwashing behavior were also able to decrease transmission. The type and order of interventions are important in successfully attenuating disease risk, with infrastructural and engineering controls being most effective when administered first, followed by behavioral controls after successful attenuation of sufficient alternate transmission routes. Beyond COVID‐19, our modeling framework can be extended to address water, sanitation, and hygiene measures targeted at a range of environmentally mediated infectious diseases.

## 1 INTRODUCTION

During the COVID‐19 pandemic, efforts to control the transmission of SARS‐CoV‐2 focused on reducing high‐risk exposure events. In general, interventions were divided among those targeted at the individual level, through increased hand hygiene and mask‐wearing, and at the community level, through social distancing (CDC, 2022). However, these interventions rely on compliance to attain meaningful effectiveness, which may lead to mixed results in real‐world settings. In contrast, infrastructural changes have the potential to yield sustainable health benefits by structurally limiting exposure. In low‐ and middle‐income countries (LMICs), water, sanitation, and hygiene (WASH) infrastructure comprise essential services that are frequently shared due to resource constraints. Shared sanitation has long been recognized as a risk factor for enteric pathogen transmission and may also serve as a high‐risk context for respiratory pathogens such as SARS‐CoV‐2 (Caruso & Freeman, 2020) due to the ability of respiratory pathogens to persist in aerosols for hours to days. This potential for indirect contact between shared sanitation users is understudied. In this study, we elucidate the mechanisms that drive this potential risk using a transmission modeling approach and assess the effectiveness of infrastructural and behavioral mitigation strategies from a hierarchy of control framework. We use SARS‐CoV‐2 as an example respiratory pathogen due to its impact as a pandemic strain.

Prior studies have estimated that 32.5% of individuals in sub‐Saharan Africa use shared sanitation facilities—with 7.8% sharing with more than five households (Fuller et al., 2014; Heijnen, Rosa et al., 2014). Similarly, 22.4% (5.5% more than five households) of individuals in Southeast Asia were estimated to use shared sanitation facilities. When individuals rely on sanitation facilities shared by multiple households, the risk for enteric pathogen fomite transmission (and other sanitation‐related risks) may increase in some contexts, potentially due to lack of access and poor maintenance and cleaning. Although this relationship is also understudied, two studies found a weak positive association between shared sanitation use and the risk of diarrheal disease (Baker et al., 2016; Fuller et al., 2014). The findings of these empirical works were also supported by a mathematical modeling study on the impact of shared sanitation on diarrheal disease (Just et al., 2018). Despite these challenges, shared facilities have been considered an important factor in expanding sanitation coverage (Rheinländer et al., 2015), especially in urban settings (World Health Organization, 2016). Many types of shared facilities are used as the primary sanitation for individuals—including in some cases facilities in churches or schools. Facilities can be privately shared between many households in a compound or publicly shared in a neighborhood and, at times, can function as a small business. There continues to be considerable debate on the importance of shared sanitation in enteric disease transmission (Heijnen, Cumming, et al., 2014), largely due to studies reporting increased enteric disease risk associated with shared facilities (Fuller et al., 2014; Heijnen, Cumming, et al., 2014).

**FIGURE 1:**
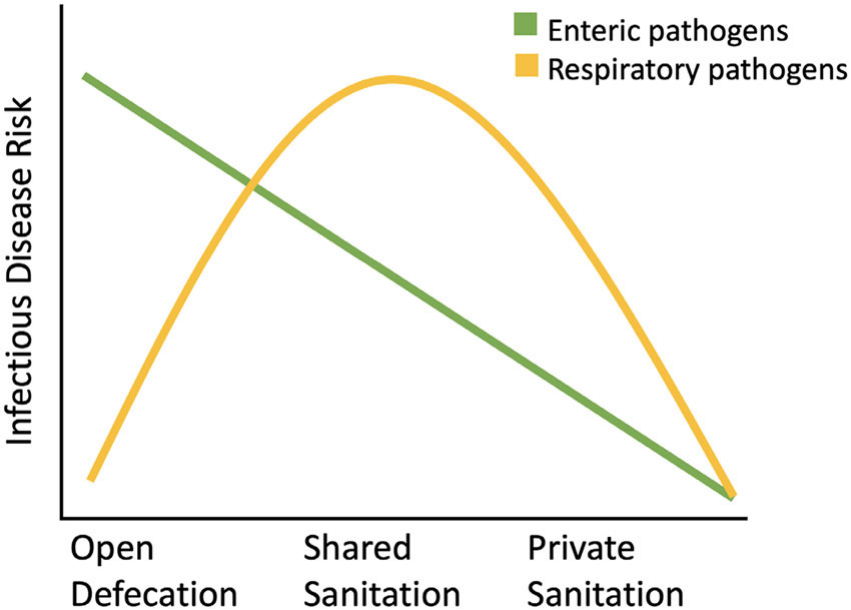
Visualization of a hypothetical interaction between sanitation infrastructure and disease risk by mode of pathogen transmission. Behavioral dynamics may add complexity to respiratory dynamics with the highest risk of disease peaking at intermediate sanitation infrastructure.

The interaction between sanitation infrastructure and disease risk may depend on the mode of pathogen transmission. Although shared sanitation facilities represent higher risks compared to private sanitation, they are an infrastructural improvement over open defecation; however, we hypothesize that for respiratory pathogens, shared facilities may result in elevated exposure risk due to high demand and throughput (Figure 1). Direct contact between users in shared sanitation facilities is rare, but indirect contact may be facilitated by the ability of respiratory viruses to remain viable in aerosols for hours to days. The magnitude of this indirect contact will increase as the number of households sharing increases, which can range from as few as five households (Antwi‐Agyei et al., 2020; Simiyu et al., 2017; Ssemugabo et al., 2021; Tumwebaze et al., 2013) to as many as 100 households (Günther et al., 2012). Latrine sharing highlights the interaction between resource constraints and infectious disease risk. Women, in particular, may be at increased risk due to higher frequency of sanitation facility use resulting from menstrual hygiene and providing assistance to family members (Caruso et al., 2017).

Shared sanitation is an important stage in developing sustainable WASH facilities, helping to bridge the gap between unimproved (e.g., open defecation) and improved options from an infrastructural perspective (e.g., private latrines that separate excreta from human contact). However, the potential for shared sanitation to facilitate transmission of respiratory pathogens, like SARS‐CoV‐2, results in the need to evaluate the risks associated with shared sanitation to facilitate the development of prevention and control measures to improve preparedness for future outbreaks.

We developed a stochastic, individual‐based model that explicitly describes exposure due to aerosol and fomite pathways in shared latrines. This model is parameterized using empirical pathogen and exposure data for SARS‐CoV‐2. Our analysis extends the traditional Quantitative Microbial Risk Assessment framework by including community and latrine transmission to evaluate the impact of feedback between transmission mechanisms. The lack of empirical data surrounding shared sanitation risk and respiratory pathogen transmission underscores the utility of a transmission modeling approach. Our approach is also widely applicable for further modeling of transmission in shared spaces for both respiratory and enteric pathogens.

## 2 MATERIALS AND METHODS

**FIGURE 2:**
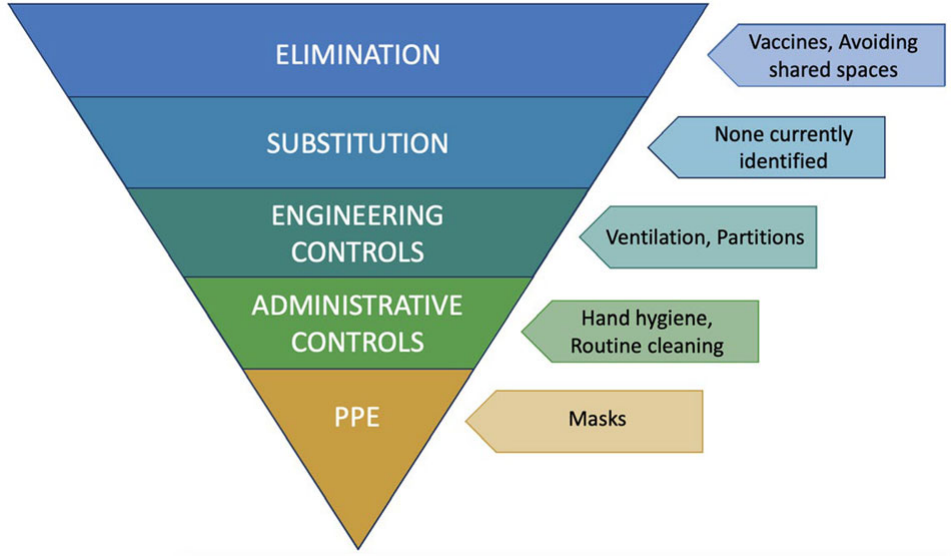
Hierarchy of controls as applied to COVID‐19 control strategies. *Source*: Adapted from National Institute for Occupational Safety and Health (NIOSH) guidelines (Cornell University, n.d.).

We conducted a simulation study to estimate the impact of shared sanitation on respiratory pathogen transmission in shared sanitation‐dependent populations. We derived parameter values corresponding to SARS‐CoV‐2 from existing literature and applied them to a novel individual‐based stochastic simulation model. We examined the risk mitigation potential of interventions at three levels of the Hierarchy of Controls—a framework originating in the occupational health field that categorizes prevention strategies based on the tradeoff between effectiveness and costs (e.g., monetary or labor) (Figure 2). In the context of pandemic response activities, interventions at the top of the Hierarchy of Controls include large‐scale, resource‐intensive strategies, such as mass vaccination and lockdown policies. Infrastructural changes, like increased latrine ventilation, rank below elimination strategies but retain the ability to systematically reduce risk by physically or mechanically isolating individuals from the pathogen. Behavioral changes in the form of mask‐wearing and hand hygiene make up the bottom tier of controls due to their reliance on high degrees of compliance to be effective. Specifically, we examined increased access to private latrines as an elimination control, latrine ventilation as an engineering control, and hand washing after latrine use as an administrative control.

### 2.1 Model parameterization

We estimated reasonable values for parameters in our simulation model derived from available literature. Our literature search was conducted using Google Scholar and Pubmed; secondary sources were evaluated using the snowball method in Scopus. Our search focused on compiling evidence‐based sources to inform (1) virus‐specific transmission parameters, including viral shedding, virus die‐off, dose–response, and transfer efficiency; (2) physiological parameters, including breathing and coughing rates; (3) behavioral parameters, including arrival/use rates, touching/inoculation rates, and handwashing frequency; and (4) infrastructural parameters, including ventilation rates, latrine volume, and accessible latrine surface area. In cases where the literature was sparse, we made assumptions based on our understanding of the biological/behavioral process. The effects of these assumptions on modeling outcomes were rigorously tested through model sensitivity analysis.

### 2.2 Model

We developed an individual‐based, stochastic simulation model to estimate the risk of SARS‐CoV‐2 transmission due to environmental contamination (air and fomites) within latrine structures as well as due to community transmission. This model can be broken down into a component representing latrine transmission and a component representing community transmission. In the latrine transmission component, latrine users that are infectious contaminate the latrine air, and fomites and latrine users that are susceptible are exposed to the contamination via inhalation and physical contact with latrine surfaces. We assume that a variable fraction of the population depends on shared latrines. However, regardless of the fraction of the population dependent on shared latrines, we assume that all individuals may use shared latrines incidentally (e.g., public toilets at community venues) based on their location or convenience (Figure 3A). Simulated individuals are assigned properties, including their disease status, the amount of viral contamination on their hands, and their average frequency of using the shared latrine. Each latrine is modeled as a single‐stall enclosed space with a fixed interior volume (liters) and ventilation rate (air changes per hour [ACH]). During a simulation, the amount of viral contamination present in the air as suspended aerosol particles and on surfaces in each latrine is tracked. Infectious individuals may shed viruses in latrines either by breathing or coughing. Of droplets released by coughing events, we assume that a fraction settles on surfaces and the remainder becomes aerosolized based on estimates in the literature of the distribution of droplet sizes released during coughs. We assume that all droplets released by breathing are aerosolized. In each case, we do not explicitly model the dynamics governing droplet size change after release. Pathogens in droplets and aerosols die off according to an exponential decay process. Aerosol particles are removed by ventilation, contributing to the overall pathogen decay rate. Uninfected individuals may inhale aerosolized virus particles or inoculate membranes (eyes, nose, or mouth) through touching contaminated surfaces while using latrines. We use a queueing process to simulate individuals arriving and waiting at the latrine (Figure 3A) and a continuous time Markov simulation to model shedding and infection occurring, whereas individuals use latrines (Allen, 2012). The community component implements a stochastic, individual‐based Susceptible, Infected, Recovered model to represent transmission from venues other than latrines (Kermack et al., 1927; Vynnycky & White, 2010). An individual's probability of infection is governed by an exponential dose–response function. We use the dose–response constant to scale viral copy ingested to viable viral copy. Infected individuals progress to the infectious stage in the community regardless of their source of infections. Infected individuals may then recover, retaining immunity against subsequent infections. A schematic illustration of the model is shown in Figure 3B. We simulate outbreak periods on the order of several months and so we do not include the potential for waning immunity. The full latrine/community model is simulated using the tau‐leaping method (Gillespie, 2001).

FIGURE 3(A) Flowchart diagram of latrine use simulation depicting initialization and simulation processes in our individual‐based latrine usage model. (B) Illustration of the full transmission model including movement between latrines and the community. Individuals from the community use shared latrines. Infectious individuals may shed in latrines; susceptible individuals may be exposed to pathogens via aerosols or fomites and then return to the community where they may transmit to other community members.

**Figure:**
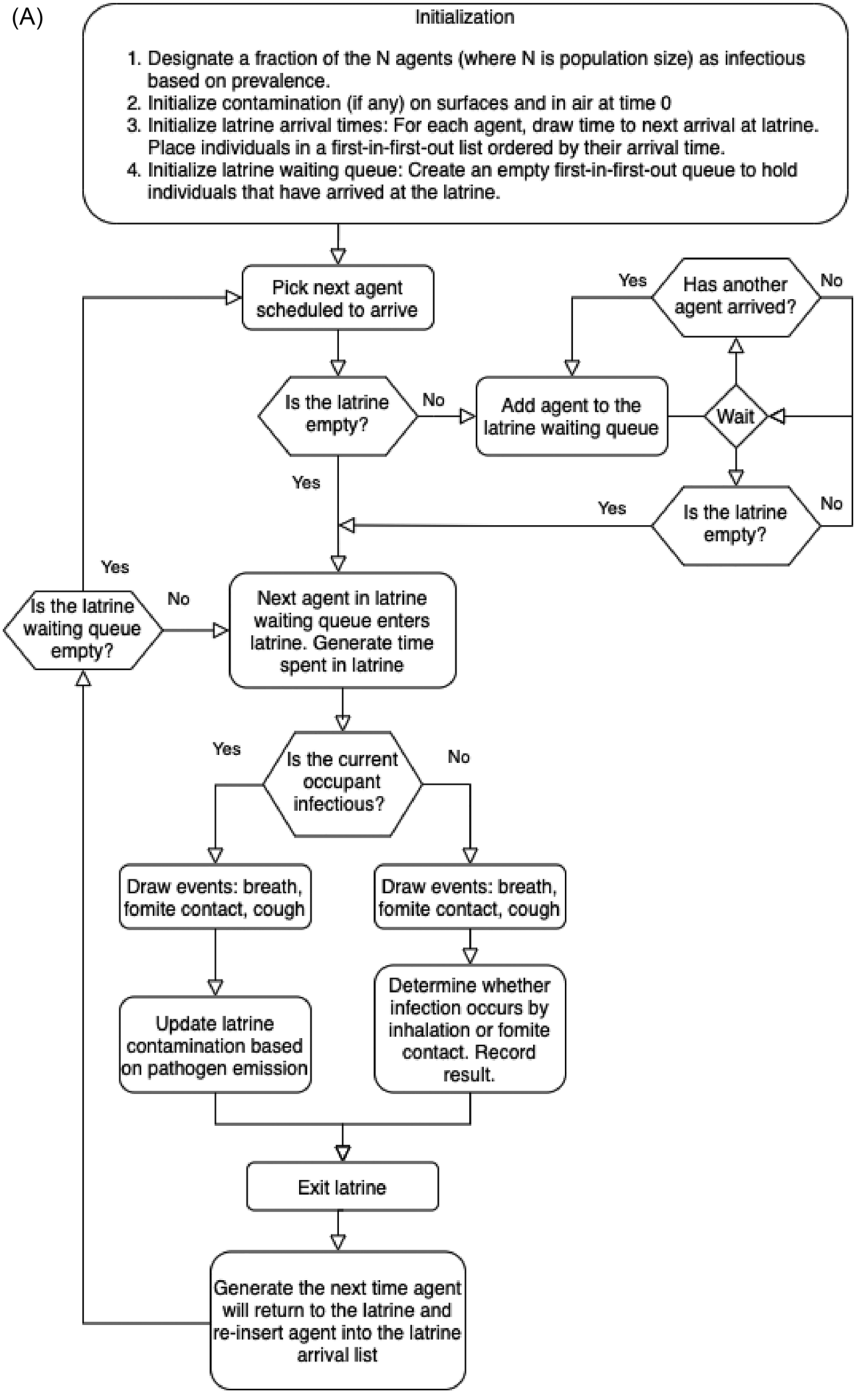

**Figure:**
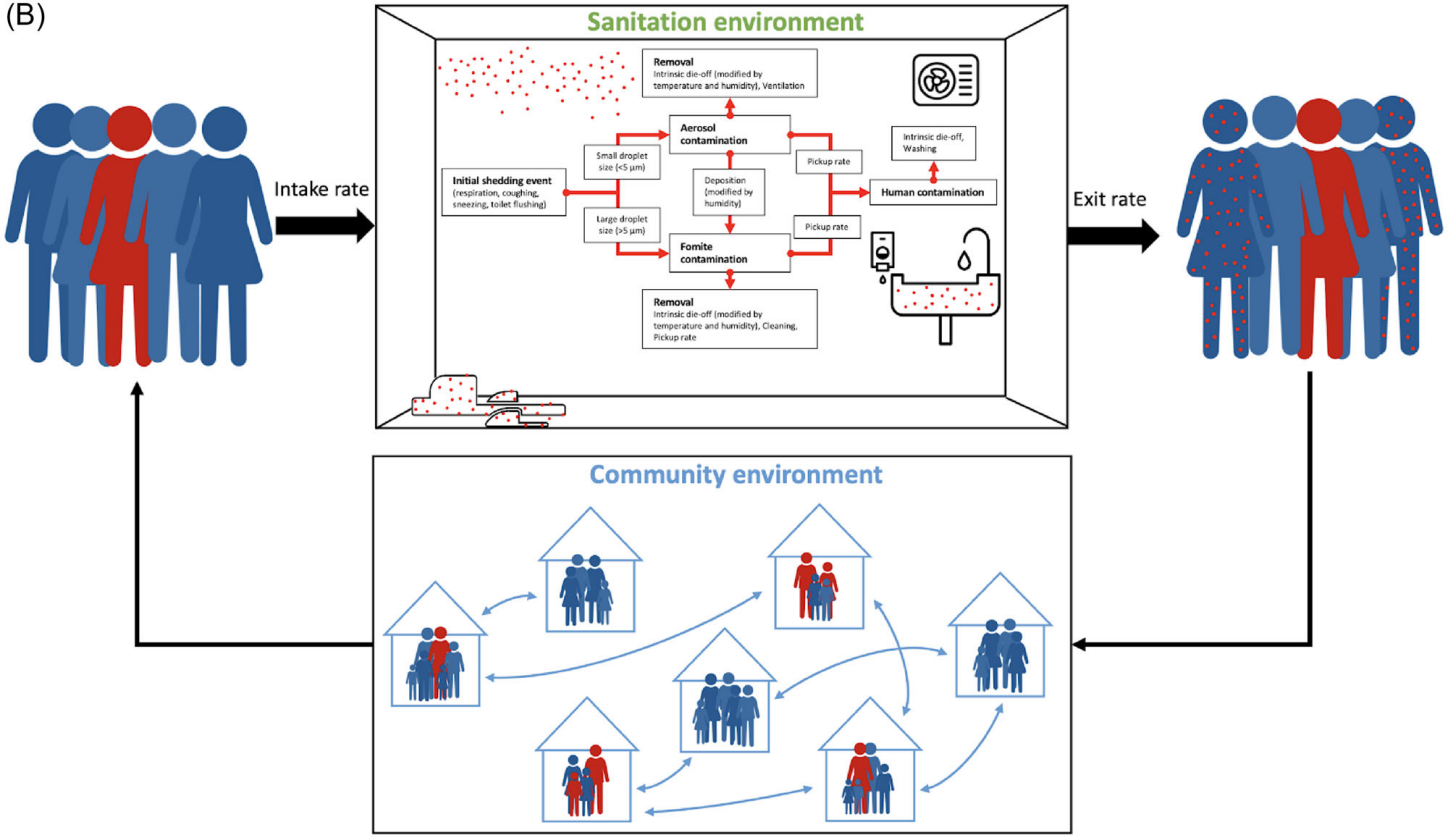

Parameter set: In total, the model contains 22 parameters (Table 1). Specific parameter values used in simulations are presented in Section 3.

**TABLE 1:** Model parameters used for simulations.

Name	Units	Value
**Incidental use arrival rate**	1/days	0.5
**Primary use arrival rate**	1/days	2
**Average latrine use duration**	Minutes	7
**Surface touch rate**	1/min	0.5
**Self‐touch rate**	1/min	0.25
**Breathing rate**	1/min	10
**Breathing volume**	L	0.5
**Breathing shedding mean**	CFU	11,772
**Breathing shedding standard deviation**	CFU	3888
**Pickup fraction**		0.1
**Latrine internal volume**	ft^3^	54
**Accessible surface fraction**		0.01
**Dose–response constant**		4.1 × 10^2^
**Coughing rate**	1/min	0.73
**Coughing shedding droplet mean**	CFU	1000
**Coughing shedding droplet standard deviation**	CFU	10
**Coughing shedding aerosol mean**	CFU	10,000
**Coughing shedding aerosol standard deviation**	CFU	100
**Aerosol die‐off rate**	1/min	0.01
**Surface die‐off rate**	1/min	0.002
**Viable copy proportion**		0.01
**Ventilation rate**	ACH	Variable
**Hand‐washing probability**		Variable
**Community transmission rate**	1/days	Variable
**Onset rate**	1/days	7
**Recovery rate**	1/days	10

Abbreviation: ACH, air changes per hour.

Analysis plan: A total of 500 simulations were performed for each combination of model parameters to establish average values for key metrics. We calculated pathway‐specific average attack rates (ARs) associated with latrine aerosols, latrine fomites, and community sources, as well as the total AR for each set of simulation parameters. We investigated the role of community and latrine‐level interventions by calculating the preventive fraction (PF) for each intervention scenario as follows: *PF* = (*AR_Baseline_*―*AR_Intervention_*)/*AR_Baseline_* where *AR_Baseline_* is the average AR for a baseline scenario without the intervention, and *AR_Intervention_* is the average AR for under intervention conditions, holding all else constant.

## 3 RESULTS

We identified values for key transmission parameters by examining existing empirical literature. These parameter values were then used in our simulation model analysis.

### 3.1 Parameter values

#### 3.1.1 Viral shedding

Our model considers two possible shedding routes: coughing and breathing. To estimate the extent of viral shedding via each route, we gathered information on (1) frequency of shedding event, (2) the volume of droplets emitted per event, and (3) the average viral load in relevant clinical samples.

##### 3.1.1.1 Coughing

A single cough yields a liquid weight of 0.07 ± 0.05 mL (Smith et al., 2020; Xie et al., 2009). Studies investigating respiratory samples of COVID‐19 patients report average viral loads of 6.76 × 10^5^, 7 × 10^6^, and 105.85 copies per mL (Kleiboeker et al., 2020; Wölfel et al., 2020) (range: 641–1.34 × 10¹¹ copies per mL, Pan 2020). We assume an average viral load of 10^6^ copies/mL to match the order of magnitude as viral titer estimates used in other COVID‐19 modeling studies (10^6^ (Riediker & Tsai, 2020), 7 × 10^6^ (Smith et al., 2020), (Wang et al., 2020), range 10^3^–10^11^ (Schijven et al., 2020)). As such, we assume that a single cough could result in shedding of 20,000–120,000 virus copies distributed between droplets and aerosols. Smith et al. (2020) reported that 98% of liquid volume from a cough is contained in large droplets (100–1000 εm), whereas 2% is contained in microdroplets. Assuming pathogen concentration is the same across all particle sizes, we find that a single cough produces 19,600–117,600 virus copies in droplets and 400–2400 virus copies in aerosols. Previous SARS‐CoV‐2 transmission modeling studies have estimated that a single cough yields 9467–5 × 10^5^ virus copies in droplets (Smith et al., 2020; Wang et al., 2020) and 333–10^4^ virus copies in aerosols (Riediker & Tsai, 2020; Smith et al., 2020; Wang et al., 2020).

##### 3.1.1.2 Breathing

Clinical testing of viral shedding in exhaled breath of COVID‐19 patients reports shedding of 10^2^–10^5^ virus copies in aerosol particles (size < 5 εm) during 30 min of breathing (or 33.3–3.33 × 10^4^ copies in 10 min) (Leung et al., 2020). Another clinical study of COVID‐19 patients reports a higher breath emission rate of 1.03 × 10^5^–2.25 × 10^7^ virus copies in 1 h (or 1.7 × 10^4^–3.75 × 10^6^ copies in 10 min) (Ma et al., 2021). 10 min of tidal breathing yields 1–3 mL of exhaled breath condensate (Horváth et al., 2005; Hunt, 2007, 2002; Soyer et al., 2006). Assuming the same viral load of 10^6^ copies/mL in all respiratory samples, 10 min of breathing would result in shedding of 1 × 10^6^–3 × 10^6^ copies of SARS‐CoV‐2 virus. This estimate is within the range reported by Leung et al. and Ma et al. We assume that all viruses emitted through breathing remain aerosolized.

#### 3.1.2 Viral persistence

##### 3.1.2.1 Surfaces

The length of survival of SARS‐CoV‐2 is heavily influenced by the surface it is on. Nonporous surfaces such as plastic and steel appear to preserve the virus for longer periods of time than rougher surfaces like clothing (Chatterjee et al., 2021; Corpet, 2021). van Doremalen et al. (2020) measured SARS‐CoV‐2 half‐lives of 5.63 (95% CI: 4.59, 6.86), 6.81 (95% CI: 5.62, 8.17), and 3.46 (95% CI: 2.34, 5.00) hours on steel, plastic, and cardboard, respectively, at 23°C and 40% RH. Gidari et al. (2021) measured SARS‐CoV‐2 half‐lives of 5.3, 4.4, and 4.2 h on plastic, stainless steel, and glass surfaces, respectively at 3–25°C and 40%–50% RH. Hirose et al. (2021) examined the fate of SARS‐CoV‐2 (DMEM) on human skin and found a median half‐life of 3.53 h (95% CI: 3.02, 4.16) coupled with a survival time of 9.04 h (95% CI: 7.96, 10.2). In addition to surface material, environmental variables like temperature and humidity have also been shown to play a significant role in SARS‐CoV‐2 persistence (Kwon et al., 2021, Ridell et al., 2020). For the purpose of this study, we did not directly consider the impact of temperature and humidity of the persistence of SARS‐CoV‐2. We use plastic surfaces as the baseline for the purpose of estimating viral persistence.

##### 3.1.2.2 Aerosols

Aerosolized SARS‐CoV‐2 viral particles seem to have lower survival success compared to surfaces with van Doremalen et al. (2020) reporting a median half‐life of 1.09 h (95% CI: 0.64, 2.64) and infectious particle residence time of at least 3 h. Fears et al. (2020) reported a longer residence time, with infectious aerosolized particles persisting for up to 16 h, but were unable to provide a biological half‐life due to a remarkably flat decay curve. Although van Doremalen et al. (2020) reported a faster decay rate of 0.66, the study's short observation time of 3 h may have only captured the initial dynamics of a biphasic decay rate that would allow for the persistence of aerosolized particles up to 16 h, as observed by Fears et al. (2020). To accommodate both results, we assume that virus particles in aerosols decay at an intermediate rate of 0.01 particles/min.

#### 3.1.3 Dose–response relationship

Due to the lack of literature on the minimum infectious dose of SARS‐CoV‐2, we use studies on SARS‐CoV to estimate this value. Watanabe et al. found a 10% response at 43 PFU (95% CI: 20,81) and a 50% response at 280 PFU (95% CI: 130,530). The dose–response curve for intranasal infection identified by these authors was *p*(*d*) = 1−exp(−*d*/*k*) where *k* = 4.1 × 10^2^ (Watanabe et al., 2010). This constant has been used in other COVID‐19 modeling studies assessing risk (Amoah et al., 2021; Pitol & Julian, 2021). Given the novelty of the SARS‐CoV‐2 virus, little is known about the differences in dose–response relationship across exposure pathways (e.g., inhalation and membrane). However, given the high infectivity of variant SARS‐CoV‐2 strains such as Delta and Omicron, the dose–response constant from SARS‐CoV is likely to be an underestimate. In particular, Prentiss et al. (2022) found that the infectious dose for SARS‐CoV‐2 is approximately 1 PFU. We use the dose–response constant for SARS‐CoV as we use the difference in magnitude compared to SARS‐CoV‐2 as a scaling factor to transform viral copies from our shedding process into viable infectious copies.

#### 3.1.4 Viral transfer efficiency

##### 3.1.4.1 Fomite → hand

Although efficiency of SARS‐CoV‐2 transfer between contaminated surfaces and hands has not been well‐characterized, previous COVID‐19 risk assessments have used different viruses to estimate SARS‐CoV‐2 transfer efficiency. A 2014 review reported a median transfer efficiency for norovirus between fomites and hands of 51% (range, 33%–68%) (Ryan et al., 2014), and this estimate was used in a COVID‐19 risk assessment (Amoah et al., 2021). Another COVID‐19 risk assessment (Pitol & Julian, 2021) used MS2 as a proxy, parameterizing their model with transfer efficiencies of 37.4% on plastic and 79.5% on metal (Lopez et al., 2013). These values are likely overestimates as they come from nonenveloped viruses and SARS‐CoV‐2 is an enveloped virus. The transfer efficiency for enveloped viruses is known to be lower than for nonenveloped viruses. As such, modeling studies that use enveloped viruses (e.g., influenza and transfer efficiency 10%) as a proxy for SARS‐CoV‐2 transfer may more accurately estimate COVID‐19 risk (Kraay et al., 2021). Given that SARS‐CoV‐2 is an enveloped virus that demonstrates significantly long persistence on skin (9–11 h) (Hirose et al., 2021), we assume a relatively higher transfer efficiency of 20% (Castaño et al., 2021).

##### 3.1.4.2 Hand → membrane

Efficiency of viral transfer during inoculation of mucous membranes (e.g., eyes, nose, and mouth) has been studied by Rusin et al. (2002) who reported a 33.9% transfer rate of phage PRD‐1 between the fingertips and mouth. Pitol et al. (2021) studied skin‐to‐saliva transfer efficiency of nonenveloped MS2 and Qβ and enveloped Φ6 phage, reporting transfer efficiencies of 58.3% ± 14.8% and 20.1% ± 6.3% depending on the moisture of skin inoculum during transfer. We assume an inoculation transfer efficiency of 33% and an inoculation rate of 1 touch per 4 min, a rate slightly lower than the average rate for face contact due to the latrine environment (Pitol & Julian, 2021).

#### 3.1.5 Breathing and coughing rates

Current estimates report a normal respiration rate of 12–16 breaths per minute (Johns Hopkins University, 2022) and a tidal volume of 450 mL (Hallett et al., 2022). We found a lack of evidence on COVID‐19 coughing rate in the literature; however, one study, including 17 COVID‐19‐positive patients, observed on average 17 coughs during 30 min of exhaled breath collection (Leung et al., 2020). We assume that coughing in COVID‐19 patients is less frequent than coughing observed in influenza A and B patients (2.5–19.5 coughs every 30 min) (Yan et al., 2018), but higher than coughing frequency observed in healthy patients (0.3875 coughs every 30 min) (Yousaf et al., 2013). Overall, coughing is present in 76% of COVID‐19 cases (Huang et al., 2020).

#### 3.1.6 Latrine use patterns

Based on biologically driven urination and defecation frequencies, we assume individuals may use the latrine 3–4 times per day (Heaton et al., 1992; Lukacz et al., 2009; Wrenn, 1990). In a private shared facility, where 20 individuals may share a latrine (Antwi‐Agyei et al., 2020; Simiyu et al., 2017; Tumwebaze et al., 2013), this waste elimination frequency corresponds to a use rate of one user every 9–12 min for private shared latrines. In contrast, public shared facilities may service larger groups of 150 individuals; however, these populations may distribute their sanitation needs across several alternatives (open defecation/urination, private facilities) leading to a lower use frequency of 1–2 times per day (The World Bank et al., 2014). This corresponds to a use rate of one user every 2.4–4.8 min for public share latrines.

#### 3.1.7 Ventilation

Although information on ventilation rates inside public latrines is limited, our results are contextualized by Dumpert et al.’s (2009) study reporting an average air exchange rate of 2.4 ACH in a public ventilated improved pit (VIP) latrine located in Ghana. Although this study provides an empirically grounded benchmark, we assume that the limited sanitation facilities of interest may have both poorer ventilation (e.g., concrete stalls) and superior ventilation (e.g., doorless structures). Importantly, the impact of improved or worsened ventilation conditions around a ventilation rate of 2.4 ACH remains significant, indicating that changes in ventilation infrastructure could play an important role in infection control in the shared sanitation setting.

### 3.2 Simulation model analysis

#### 3.2.1 Impact of reducing latrine sharing

**FIGURE 4:**
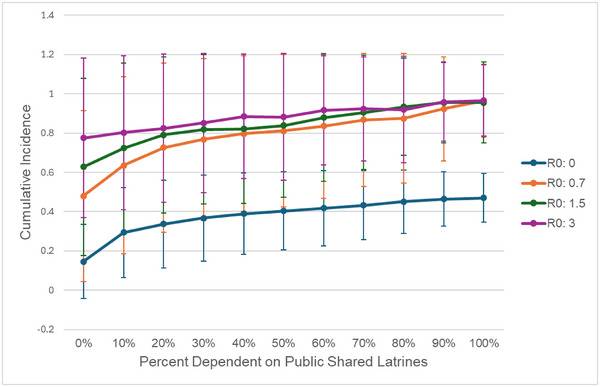
The impact of dependence on public shared latrines on COVID‐19 cumulative incidence for a range of community transmission rates (*R*_0_ indicates the reproduction rate from community transmission specifically). Simulations are conducted with a ventilation rate of 2.4 air changes per hour (ACH). Error bars indicate the standard deviation for each cumulative incidence point across 500 simulations.

We use our model to examine changes in community‐level infection risk while varying the fraction of the population who depend on shared sanitation as their primary facility—either publicly or privately shared. Cumulative incidence decreases as the fraction of the population dependent on shared latrines decreases. This trend is characterized by significant nonlinearity. For example, reducing the dependence on shared sanitation from 100% to 80% in a community with *R*_0_ = 1.5 and a latrine ventilation rate = 2.4 ACH (Figure 4) yields a PF of 3.1% (CI decreases from 0.96 to 0.93). However, the same 20 percentage point reduction in shared sanitation dependence in the same community from a baseline of 20%–0% yields a PF of 20% (CI decreases from 0.79 to 0.63).

Reduction in shared latrine use consistently attenuates disease spread; however, the magnitude of this effect depends on background community transmission. When community transmission is high, the relative importance of transmission in the sanitation environment is reduced, which attenuates the benefit of latrine‐level interventions (Figure 4). When the community *R*_0_ is 3 with a latrine ventilation rate of 2.4 ACH and there is 100% dependence on shared latrines, latrine transmission accounts for 87% of all infections but eliminating all dependence on shared sanitation yields a PF of 20% (Figure 4, purple line). In contrast, when the community *R*_0_ is 0.7 (i.e., when community‐level interventions would have otherwise mitigated transmission) with the same ventilation rate, infections due to 100% latrine sharing account for 95% of the total transmission; reducing to 0% sharing has a much stronger impact, with a PF of 51% (Figure 4, blue line).

#### 3.2.2 Impact of latrine ventilation

**FIGURE 5:**
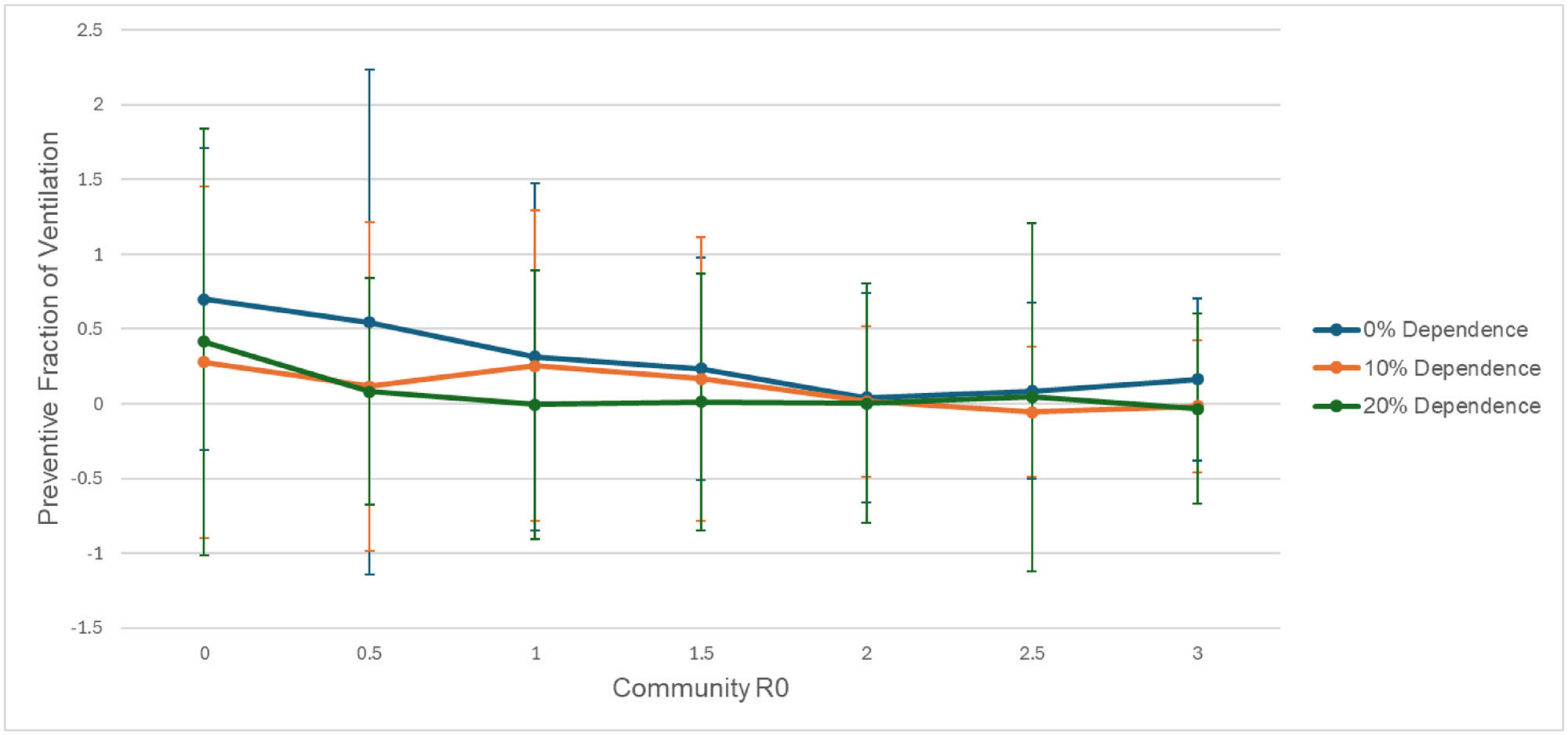
The preventive fraction (relative reduction in risk attributable to the intervention) of increasing latrine ventilation from 0.25 air changes per hour (ACH) to 4 ACH for a range of community transmission rates and four levels of dependence on shared latrines. Error bars indicate the standard deviation for each cumulative preventive fraction across 500 simulations.

We investigated the impact of latrine ventilation on COVID‐19 risk by testing a range of ventilation rates contextualized by real‐world settings including a concrete enclosure with poor ventilation (0.25 ACH), a VIP latrine in Ghana (2.4 ACH) (Dumpert et al., 2009), and a well‐ventilated US classroom or residence (4 ACH) (US EPA, 2019). For the purpose of intervention simulations, we compared a baseline ACH of 0.25 to an intervention level of 4 ACH. Our model predicts that increased ventilation has the potential to substantially decrease the risk of latrine‐associated infection (Figure 5). Importantly, the potential for ventilation to attenuate latrine‐associated risk is sensitive to both community *R*_0_ and dependence on shared latrines. As the community *R*_0_ decreases the benefits of ventilation increase. For example, PF = 16% when community *R*_0_ = 3 for 0% dependence on shared latrines compared to PF = 70% when community *R*_0_ = 0 at the same level of dependence on shared latrines (Figure 5). When community *R*_0_ is 0, the benefit of increasing ventilation increases with decreasing dependency on shared latrines (Figure 5).

**FIGURE 6:**
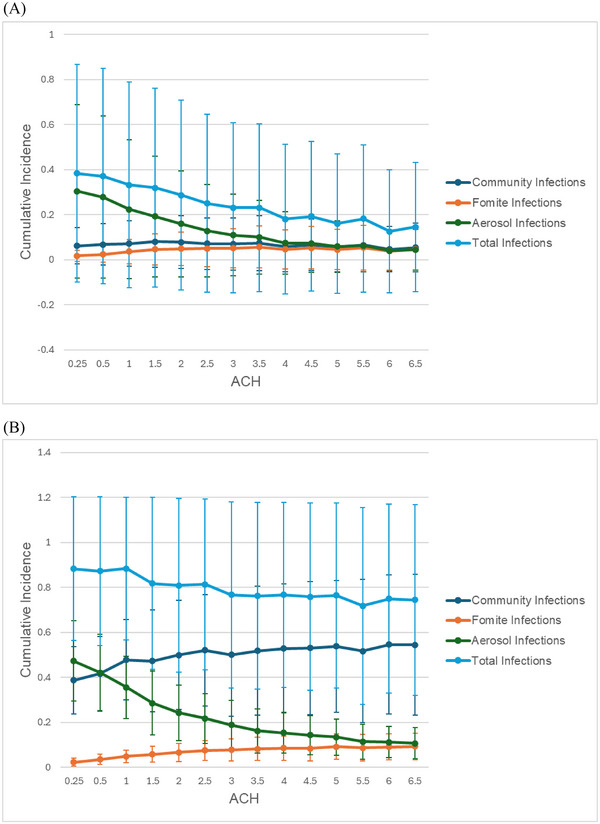
COVID‐19 incidence by transmission pathway at 0% dependence on public latrines as latrine ventilation increases. (A) Community transmission is low (*R*_0_ = 0.7). (B) Community transmission is high (*R*_0_ = 3). Error bars indicate the standard deviation for each cumulative incidence point across 500 simulations.

When community *R*_0_ and dependence on shared latrines are both low (community *R*_0_ = 0.7, 0% dependence), alternate routes of transmission (e.g., community transmission) are sufficiently attenuated, allowing ventilation to significantly reduce aerosol and total incidence (Figure 6A). Under these ideal conditions, improving ventilation from 0.25 to 4 ACH can lead to a PF of about 52% (Figure 5). When community *R*_0_ is increased (community *R*_0_ = 3, 0% dependence), the positive impact of improved ventilation is subject to diminishing returns due to the redirection of aerosol transmission potential to alternative competing pathways. Under these conditions, any reductions in aerosol incidence are taken up by community transmission, leading to a constant level of total incidence across a wide range of ventilation rates (Figure 6B). This competing risk phenomenon significantly reduces the PF of ventilation when community *R*_0_ is high (Figure 5).

#### 3.2.3 Impact of hand washing

When community transmission is attenuated, and a large proportion of transmission occurs in the sanitation environment, we observe that improved ventilation conditions are an effective intervention for reducing transmission (Figure 6A). However, these reductions are subject to diminishing returns when community transmission is high due to competing risks between the attenuated aerosol pathway and the fomite pathway which is able to replace aerosol infections as an alternative transmission route and sustain transmission. As a result, even with higher ventilation rates, total incidence converges to the combined contributions of fomite infection and aerosol infection (Figure 6B).

**FIGURE 7:**
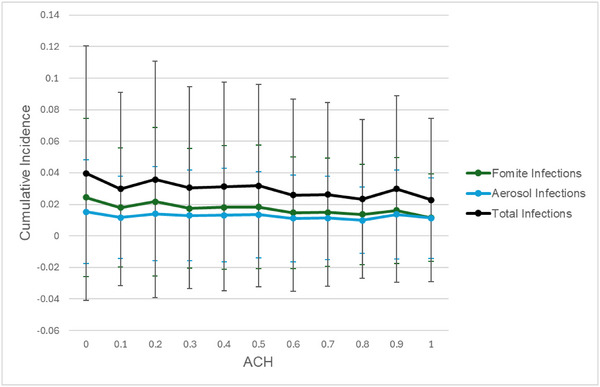
Attack rate by source for increasing hand‐washing compliance after the use of a shared latrine. In this scenario, community transmission is set to a low level (community *R*_0_ = 0.7), 20% of the population is dependent on shared latrines, and the ventilation level of latrines is set to 4 air changes per hour (ACH). Error bars indicate the standard deviation for each cumulative incidence point across 500 simulations. Note that the scale of the *y*‐axis is an order of magnitude below other figures.

Under conditions of minimal community transmission and substantially improved ventilation (ACH = 10), therefore, interventions targeting the fomite pathway may represent the final step in attenuating transmission in the latrine environment. When latrine aerosol transmission, community transmission, and dependence on public shared latrines are low due to the combined impact of ventilation, community‐level mitigation measures, and private latrines, our model predicts that hand washing after latrine use is effective at reducing the remaining cumulative incidence (PF = 42% comparing perfect hand‐washing compliance to none) (Figure 7, black line).

## 4 DISCUSSION

Our analysis suggests that shared sanitation facilities such as public latrines may act as transmission venues for infectious respiratory diseases such as COVID‐19. Like many other infectious respiratory viruses, SARS‐CoV‐2 exhibits a high degree of shedding and can persist in aerosols and on surfaces for hours to days; latrines in LMICs are relatively small with poor ventilation and high use patterns. Our simulations reinforce the potential for the proportion of individuals who rely on shared sanitation as their primary sanitation option to amplify or drive community outbreaks.

We analyzed interventions at different levels of the hierarchy of controls including reducing dependence on shared latrines, improving latrine ventilation, and improving hand‐washing compliance after latrine use. We found that removing dependence on shared latrines had a substantial independent effect on respiratory disease transmission, especially when community transmission was high. Improving latrine ventilation had a smaller independent effect but a larger impact when shared sanitation and community transmission were already decreased. Hand‐washing compliance had the smallest independent impact but did yield reductions in transmission in scenarios where all other transmission routes had been attenuated. The priority intervention for mitigation is related to the background community rate of infection.

### 4.1 Characterizing latrine use and transmission

The potential for respiratory disease transmission in public shared latrines is the product of the design and engineering of latrine, pathogen, and human factors such as latrine usage patterns and hygiene compliance. Latrine design determines factors such as the total volume of each toilet stall, the position of contact surfaces within and around the latrine, and the effective ventilation rate of the latrine. In particular, latrine ventilation can vary considerably and may not necessarily reflect the quality of a latrine from sanitation, public sewage, or design perspective. For example, a VIP latrine—as they have little in‐chamber air exchange—may exhibit a ventilation rate of 2.4 ACH [cite], whereas an unenclosed latrine would have a substantially higher effective air exchange rate.

Pathogen‐specific factors such as shedding, air and surface persistence, and dose–response determine the degree of contamination present in a latrine as well as the likelihood of infection given exposure to latrine contamination. For SARS‐CoV‐2, a substantial amount of virus is released both by coughing and breathing. Although the majority of shed viral copies are nonviable, the amount of viable copies is generally still more than sufficient to infect many additional individuals. Persistence on surfaces can range from hours to days (Gidari et al., 2021; Hirose et al., 2021; Kwon et al., 2021; Riddell et al., 2020; van Doremalen et al., 2020), whereas aerosolized viruses can persist on the order of hours (Fears et al., 2020; van Doremalen et al., 2020), mediated by the ventilation rate of a given space. Combined with a relatively low infectious dose (Amoah et al., 2021; Pitol & Julian, 2021; Watanabe et al., 2010), these properties are reflected in the rapid spread of COVID‐19 outbreaks worldwide.

Latrines represent a particularly important venue from a behavioral perspective. Urination and defecation are biological necessities, rendering sanitation infrastructure essential. Both urination and defecation occur at relatively regular intervals (Heaton et al., 1992; Lukacz et al., 2009; Wrenn, 1990), potentially requiring multiple uses of a given latrine throughout the day. This creates the potential both for recontamination of a latrine by an infectious individual as well as multiple opportunities for any given susceptible individual to become infected in a latrine. Taken together, these aspects suggest that latrines cause more transmission than would otherwise be expected from a venue that is used individually and sequentially, as opposed to large venues that are generally considered superspreading sites (e.g., churches or schools). Shared sanitation is often relied upon by the poorest urban residents; those who might already live in high‐density households, meaning infecting these family units could lead to super‐spreader events among individuals with poor access to healthcare.

### 4.2 Mitigating latrine transmission

Our intervention scenario simulations support the potential role of shared latrines in SARS‐CoV‐2 transmission and highlight the importance of transitioning to well‐designed private household latrines. Eliminating the dependence on shared latrines has the largest independent protective effect of the three interventions we examined. However, the impact of marginal reductions in shared latrine dependence is more nuanced. Reducing (but not eliminating) shared latrine dependence has a larger protective effect at low levels of latrine dependence than at high levels of latrine dependence. These results suggest that infrastructure projects targeting sanitation improvements are likely to attenuate respiratory disease outbreaks, but planners may need to consider local conditions in order to accurately gauge the degree of investment needed to attain the desired degree of health gains.

Although less effective as an independent intervention, we found that improving latrine ventilation still yielded a meaningful protective effect and complemented reductions in shared latrine dependence. Mechanistically, ventilation reduces pathogen persistence by removing infectious aerosols from the latrine space. This process should occur at a high enough rate to reduce aerosol contamination within the latrine between uses such that susceptible users do not receive a sufficient infectious dose after the latrine is contaminated by a previous infectious user. In practice, this is difficult to achieve when shared latrines are subject to high demand. Once a community has transitioned to private latrines, our model assumes that individuals use shared latrines only for convenience of location. In this scenario, improved latrine ventilation at a rate of 4 ACH is able to act as desired.

Improving hand‐washing compliance has the smallest independent protective effect of the three interventions we simulated. This is likely because latrine aerosol transmission acts as a stronger transmission pathway than latrine fomite‐mediated transmission due to more frequent exposure events through breathing compared to physical contact with latrine fomites. We would expect this to be different had we modeled enteric pathogens that exhibit higher relative fomite transmission. The only scenario in which we observed a substantial protective effect from hand washing was when community transmission and dependence on shared latrines were low and latrine ventilation was high. In this case, fomite‐mediated transmission was the only remaining transmission pathway. However, by comparison to our other simulated interventions, hand washing requires consistent adherence, which is rarely demonstrated empirically, even when strong hand‐washing policies are in place (Allwood et al., 2004; Borchgrevink et al., 2013; Ford et al., 2014).

An important feature of disease transmission modeling is the ability to examine the impact of and interactions between multiple transmission pathways. In our model, feedback between community, latrine aerosol, and latrine fomite transmission played a major role in determining the effectiveness of interventions. As infectious cases in the community rise, they contaminate shared latrines more frequently. Cases caused by infections in shared latrines spend the majority of their time at community sites, amplifying community transmission. Latrine‐associated infections may occur due to either aerosol or fomite exposure. As a result, in some scenarios, reductions in transmission from single‐pathway interventions (e.g., reducing dependence on shared latrines without modifying other conditions) are compensated for by an increase in transmission from another pathway (e.g., community transmission). This phenomenon occurs as long as the total transmission potential between community and latrine sources is sufficiently high. Competing risks also impact the effectiveness of ventilation and hand washing. Increasing the air exchange rate in shared latrines only yields a substantial reduction in cumulative incidence when transmission from the community has been attenuated and few individuals are dependent on shared latrines. Similarly, even high adherence to hand washing after latrine use is most effective once all other pathways have been addressed.

### 4.3 Limitations and future work

Our transmission model provides a mechanistic framework to evaluate the role of shared sanitation in respiratory disease outbreaks. However, several aspects of the system have been simplified to improve simulation performance and reduce the overall complexity of analysis. Community transmission venues are represented by a single mass‐action process in our model. This is likely to be a reasonable approximation of the dynamics of highly infectious pathogens such as SARS‐CoV‐2 but may require additional heterogeneity to capture less infectious pathogens. We also assume that the rates of events in our model are homogeneous with respect to time (e.g., latrine usage rates). This is a common approach in disease transmission modeling studies, though more realistic daily schedules or changes in behavior in response to outbreaks could impact the observed effect of interventions. The validity of our simulation results also relies on the specific parameter values chosen. The dose–response curve for SARS‐CoV‐2, shedding volume, and other aspects vary by strain and across pathogens. Environmental factors such as temperature and humidity impact both viral persistence and the dynamics of respiratory droplets and aerosols. We did not implement these factors to focus on a more generic environment, but future work may be needed to clarify these effects. Finally, our model only considers one type of latrine, a single‐stall enclosed structure. Future work may extend this model to include multi‐stall structures, as these may lead to increased transmission due to simultaneous use. In addition, it may be important to consider the impact of shared sanitation on both enteric and respiratory disease transmission.

## 5 CONCLUSIONS

Our model framework and simulation results can provide heuristic guidance for WASH intervention planners for future respiratory disease outbreaks. Where they are feasible, infrastructural changes such as transitioning to private latrine facilities are likely to offer the greatest benefit in terms of mitigating respiratory disease outbreaks. Although costly, these measures would also have a sustained impact, as opposed to shorter term interventions. Engineering controls such as designing latrines for higher ventilation rates aimed at removing respiratory aerosols could be considered a supporting measure, with a similar sustained impact. Finally, behavior changes such as encouraging or mandating hand washing appear least likely to substantially reduce respiratory disease transmission in latrines but are relatively low cost and may yield larger benefits for mitigating enteric disease transmission. A similar modeling approach to understand the impact of shared sanitation on enteric pathogens would contribute further to the debate on the risks posed by shared sanitation (Fuller et al., 2014).

## AUTHOR CONTRIBUTIONS

Joseph N. S. Eisenberg and Matthew C. Freeman conceived the study aims and acquired funding. Joseph N. S. Eisenberg, Matthew C. Freeman, Michael A. L. Hayashi, Hannah Van Wyk, Mondal Hasan Zahid, Sophia M. Simon, and Kaiyue Zou jointly developed study methodology. Michael A. L. Hayashi, Hannah Van Wyk, Kaiyue Zou, and Sophia M. Simon curated data sources and Michael A. L. Hayashi, Hannah Van Wyk, Mondal Hasan Zahid, Kaiyue Zou, and Sophia M. Simon conducted formal analysis. Joseph N. S. Eisenberg, Matthew C. Freeman, Michael A. L. Hayashi, Hannah Van Wyk, and Sophia M. Simon managed project supervision and administration. Michael A. L. Hayashi, Hannah Van Wyk, Mondal Hasan Zahid, Kaiyue Zou, and Sophia M. Simon conducted programming and Michael A. L. Hayashi provided computing resources and validated model code. Michael A. L. Hayashi, Hannah Van Wyk created data visualizations. Sophia M. Simon and Michael A. L. Hayashi wrote the original draft. Joseph N. S. Eisenberg, Matthew C. Freeman, Michael A. L. Hayashi, Hannah Van Wyk, Mondal Hasan Zahid, Kaiyue Zou, and Sophia M. Simon reviewed the draft and provided substantial edits.

## CONFLICT OF INTEREST STATEMENT

The authors declare no conflicts of interest.
